# Evaluation of a 3D surface imaging system for deep inspiration breath-hold patient positioning and intra-fraction monitoring

**DOI:** 10.1186/s13014-019-1329-6

**Published:** 2019-07-11

**Authors:** Vincent C. Hamming, Christa Visser, Estelle Batin, Leah N. McDermott, Dianne M. Busz, Stefan Both, Johannes A. Langendijk, Nanna M. Sijtsema

**Affiliations:** 1 Department of Radiation Oncology, University of Groningen, University Medical Center Groningen, Hanzeplein 1, 9713 GZ Groningen, The Netherlands; 2 Department of Radiation Oncology, Northwest Clinics, Alkmaar, The Netherlands

**Keywords:** Surface guided radiotherapy, Deep inspiration breath-hold, Breast cancer, Cone-beam computed tomography

## Abstract

**Purpose:**

To determine the accuracy of a surface guided radiotherapy (SGRT) system for positioning of breast cancer patients in breath-hold (BH) with respect to cone-beam computed tomography (CBCT). Secondly, to evaluate the thorax position stability during BHs with SGRT, when using an air-volume guidance system.

**Methods and materials:**

Eighteen left-sided breast cancer patients were monitored with SGRT during CBCT and treatment, both in BH. CBCT scans were matched on the target volume and the patient surface. The setup error differences were evaluated, including with linear regression analysis. The intra-fraction variability and stability of the air-volume guided BHs were determined from SGRT measurements. The variability was determined from the maximum difference between the different BH levels within one treatment fraction. The stability was determined from the difference between the start and end position of each BH.

**Results:**

SGRT data correlated well with CBCT data. The correlation was stronger for surface-to-CBCT (0.61) than target volume-to-CBCT (0.44) matches. Systematic and random setup error differences were ≤ 2 mm in all directions. The 95% limits of agreement (mean ± 2SD) were 0.1 ± 3.0, 0.6 ± 4.1 and 0.4 ± 3.4 mm in the three orthogonal directions, for the surface-to-CBCT matches. For air-volume guided BHs, the variability detected with SGRT was 2.2, 2.8 and 2.3 mm, and the stability − 1.0, 2.1 and 1.5 mm, in three orthogonal directions. Furthermore, the SGRT system could detect unexpected patient movement, undetectable by the air-volume BH system.

**Conclusion:**

With SGRT, left-sided breast cancer patients can be positioned and monitored continuously to maintain position errors within 5 mm. Low intra-fraction variability and good stability can be achieved with the air-volume BH system, however, additional patient position information is available with SGRT, that cannot be detected with air-volume BH systems.

## Summary

In this study, the accuracy of a 3D surface guided radiotherapy (SGRT) imaging system was evaluated for the positioning of deep inspiration breath-hold radiotherapy breast cancer patients. Furthermore, the SGRT system was used to evaluate the patient surface stability when breath-holds were guided by an air-volume monitoring system. Promising results indicated that the SGRT is accurate with respect to cone-beam CT, and can reliably monitor the thorax position of patients undergoing deep inspiration breath-hold radiotherapy.

## Introduction

Respiratory motion introduces a variable distance between the high-dose target and the heart for left-sided breast cancer patients. Since the heart is in close proximity to the target volume, these patients are at risk of radiation-induced cardiac toxicity [1]. Deep inspiration breath-hold (DIBH) is a technique used to increase the separation between the heart and the target, and eliminate the influence of breathing motion [2–5]. However, the inter- and intra-fraction variability of the breath-hold (BH) position can compromise the benefits of DIBH [6].

The active breathing coordinator (ABC, R3.0, Elekta AB, Stockholm, Sweden) is a commercially available system designed to guide patient’s BHs [7]. The ABC system solely supports a reproducible breathing air-volume per BH however it does not verify the thorax position. Patients can employ abdominal or thoracic breathing, and non-breathing related movement is possible, while expelling consistent volumes of air [8]. Therefore, the actual position of the thorax could still vary, while using the ABC system.

Prior to irradiation, the BH position can be verified by imaging. Generally images from either electronic portal imaging devices (EPID, 2D) or cone-beam computed tomography (CBCT, 2D or 3D) are used to verify the position of the thorax [9]. However, these modalities deliver additional radiation to the patient and are therefore sub-optimal for intra-fraction monitoring of the BH position.

A solution to this problem is provided by surface-guided radiation therapy (SGRT). SGRT uses (non-invasive) optical surface imaging to reduce localization uncertainty during irradiation. This is achieved by continuously monitoring the patient’s surface during treatment and comparing it to a reference position [10]. Using SGRT during treatment allows for less patient fixation and greater speed of setup which results in increased patient comfort [10–12]. Studies have shown that SGRT can potentially replace the use of skin markers for positioning [13, 14]. However, large positioning differences can still remain between the patients’ external surface and the internal structures on which radiotherapy treatment plans are based [15].

The goal of this study is two-fold: first, to determine the accuracy of a surface imaging system AlignRT (Version 5.0.1749, Vision RT Ltd., London, UK) for position verification of breast cancer patients in BH compared to CBCT data, registered to either the clinical target volume or to a section of the patient’s surface. Secondly, to evaluate the intra-fraction variability and stability of the thorax position in BHs guided with the ABC system for breast cancer patients using AlignRT.

## Methods

### Patient data

Eighteen consecutive left-sided breast cancer patients were included in this study. In total, 16 patients received whole breast radiotherapy (WBRT), one patient received WBRT including the axilla and one patient received WBRT including a simultaneous integrated boost. All patients received DIBH treatments. Ten patients were treated with partial volumetric modulated arc therapy (par-VMAT) while eight patients were treated with conformal tangential fields. The parVMAT technique consisted of 70% tangential open fields and a 30% VMAT contribution to optimize a homogenous dose distribution. All treatment plans were robustly planned, which was achieved by using a (skin) flash of 5 mm during optimization, to account for intra- or inter-fractional target motion [16]. One patient changed to free breathing (FB) radiotherapy after 5 fractions due to difficulties in maintaining BHs. For one patient, 5 treatment fractions were excluded from the analysis due to a broken mouthpiece.

### Breath-hold guidance

The ABC device was used to guide breath-holds. It consists of a mouthpiece connected to a spirometer (to measure air-flow) and is coupled to a balloon valve [7]. The nose is pegged to ensure any breathing passes only through the mouthpiece. All patients received ABC training prior to CT acquisition. The valve within the system is closed at 75% of the maximum inhalation volume, which was determined during training. All patients received visual feedback to help the patient achieve an adequate inhalation volume. By closing the valve, the patient experienced a forced BH (no inhale or exhale was possible).

### Imaging preparation

#### CT acquisition

All patients received an ABC-guided DIBH CT scan (Somatom-Definition AS, Siemens, Forchheim, Germany) for treatment planning preparation. The cranial-caudal range of the scan region was from the diaphragm to the mandible. The slice thickness for all CT acquisitions was 2.0 mm, whereas the in-plane resolution was 1.0 mm. All patients were scanned in the Head-First-Supine orientation. Patients were positioned with a breast-board (CIVCO Medical Solutions) where the ipsi-lateral arm was placed above the head and the contra-lateral arm was positioned on the treatment table alongside the body. The treatment reference point was marked with tattoos and radiopaque wires.

#### Setup verification & registration

Patient positioning during treatment was initially performed using skin marks. Patients were shifted to the treatment isocenter with respect to the marked reference points. Pretreatment CBCTs (Elekta Infinity™ linear accelerator, (gantry range: − 15° to 180°, 120 kV, 32.5 s, 0.4 mAs) were acquired in BH position during the first three fractions and then weekly, and were used for online position correction. Three patients received daily pretreatment CBCTs because of large variations in daily setup errors. The CBCT was acquired in two or three separate BHs as the acquisition time was too long for a single BH. Registration of the CBCT to the planning CT was performed in two ways. The first registration was the clinical match, an automatic online registration to the thoracic wall. The surgical clips marking the surgical cavity should be within 5 mm of their planning CT position. If at least one clip was > 5 mm, the patient was repositioned. This method ensures optimal target coverage while minimizing the heart dose. After the registration to the thoracic wall, any deviation between the CBCT and the planning CT at the breast surface is immediately visible. If the position of the surgical clips or the breast surface deviated from the planning CT by > 5 mm for > 2 treatment fractions, a repeat CT scan was performed to evaluate the dose distribution and the treatment plan was adapted if necessary. Resulting setup corrections were applied in 3D prior to irradiation. The second method was a manual offline registration to a section of the patient’s surface. A similar region of interest (ROI) was used for the CBCT-planning CT registration as the ROI defined in the AlignRT procedure (see below).

#### Surface imaging & registration

**Fig. 1:**
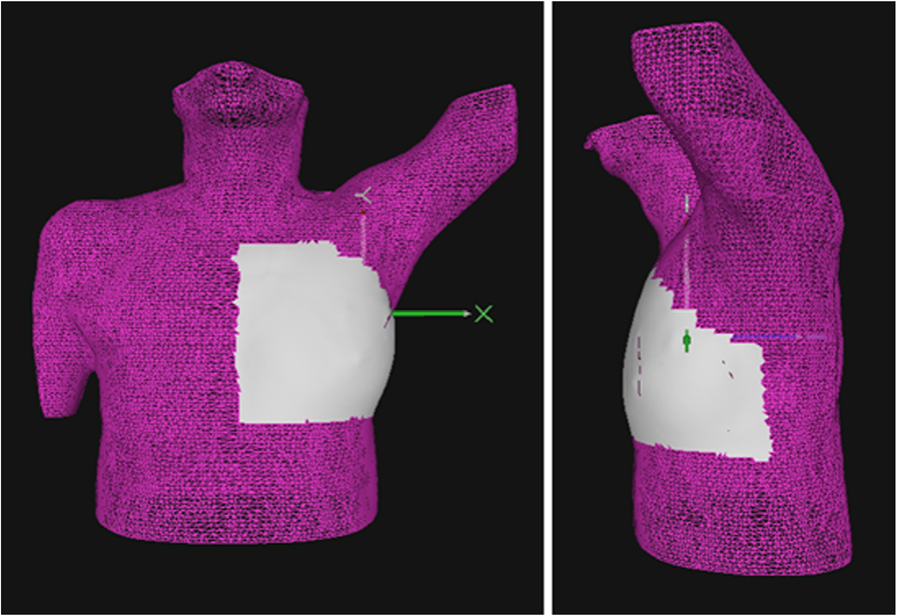
The 3D body surface of the patient from the planning CT is represented by a pink mesh. The region of interest (ROI) used for monitoring the patient surface is shown in grey

AlignRT was installed in the treatment room with a three-pod configuration. A RealTimeDelta tool, provided by VisionRT, stores the surface position deviation per frame in a text file for post-processing, for three orthogonal and three rotational degrees of freedom. A 3D body surface of the patient was generated by the planning system RayStation (Version 6.1.1.2, RaySearch, Sweden) based on the planning CT and exported to AlignRT. After importing the patient surface contour in AlignRT, the user defined a ROI for monitoring the patient’s position. The differences in each of the 6 degrees of freedom are calculated within this user-defined ROI. A standard ROI was used for every patient, as shown in Fig. 1. This ROI includes the breast with an isotropic margin of a few centimeters to ensure proper visualization of the ROI and to optimize the positioning error calculated by AlignRT. Only the left breast was included in the ROI. This has been shown to be more accurate than monitoring both breasts [17]. By using a rigid registration algorithm, AlignRT computed the deviation between the ROI and the reference surface. An average deviation is displayed for each of the six degrees of freedom. AlignRT was only used to monitor the patient during CBCT acquisition and treatment. In this study, AlignRT generated patient positioning data, hence, use of AlignRT did not influence the treatment.

### Comparison and analysis

**Fig. 2:**
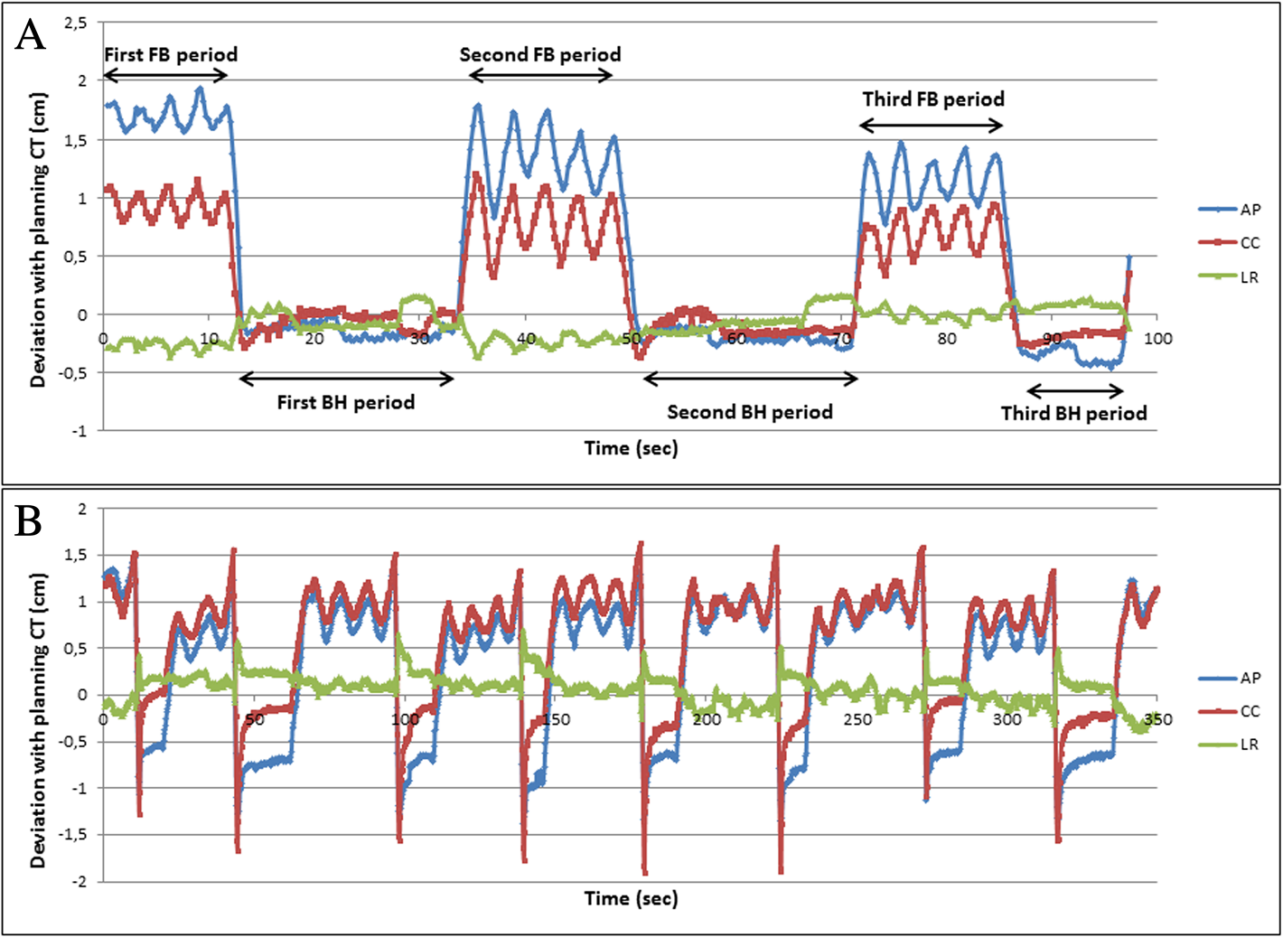
**a** Clinical AlignRT data for a BH patient during CBCT acquisition. In this case three BHs were necessary for the scan. Only the BH periods were used for setup analysis. **b** Clinical AlignRT treatment data for the BH patient with the broken mouthpiece which clearly shows stability deviations > 14 mm in the CC direction, while the air volume measured with the ABC remained constant

The AlignRT RealTimeDelta text files were imported in Excel (v2010, Microsoft) and were analyzed using an in-house macro-program. Figure 2 shows two examples of the data exported from AlignRT. Time-resolved, translational setup errors during a BH were recorded, rotations were not included.

Moreover, the BHs performed during CBCT acquisition (setup analysis) were analyzed separately from the BHs performed during treatment (intra-fraction analysis). The differences between the setup errors determined from the CBCT and AlignRT data averaged over the duration of the CBCT acquisition were determined in the three main translational directions: left-right (LR), cranial-caudal (CC) and anterior-posterior (AP). The differences were calculated in terms of the group mean (*M*), the systematic error (*Σ*), the random error (*σ*) and the 95% limits of agreement (LOA) according to the Bland-Altman analysis: *M ± 1.96* x *SD*, with *M* the mean and *SD* the standard deviation over all fractions [18]. A linear regression analysis was performed and the values for the Pearson correlation coefficients *R* were determined.

The AlignRT data during treatment was analyzed for the variability in intra-fraction position and the stability of BHs performed with the ABC system. The variability was determined from the maximum difference between the different BH levels within one treatment fraction for a patient. The stability was determined from the difference between the start and end position of each BH.

## Results

A total of 143 treatment fractions with online CBCTs for 18 patients were evaluated, with an average of 7.9 ± 3.6 CBCTs per patient. The CBCTs showed slight shading at the surface of the patient due to multiple BHs. No other artifacts in the CBCTs were observed. AlignRT was used to monitor the patient with 4–6 frames per second, depending on the size of the ROI used. The data showed no evidence of camera occlusion at any gantry angle.

**Fig. 3:**
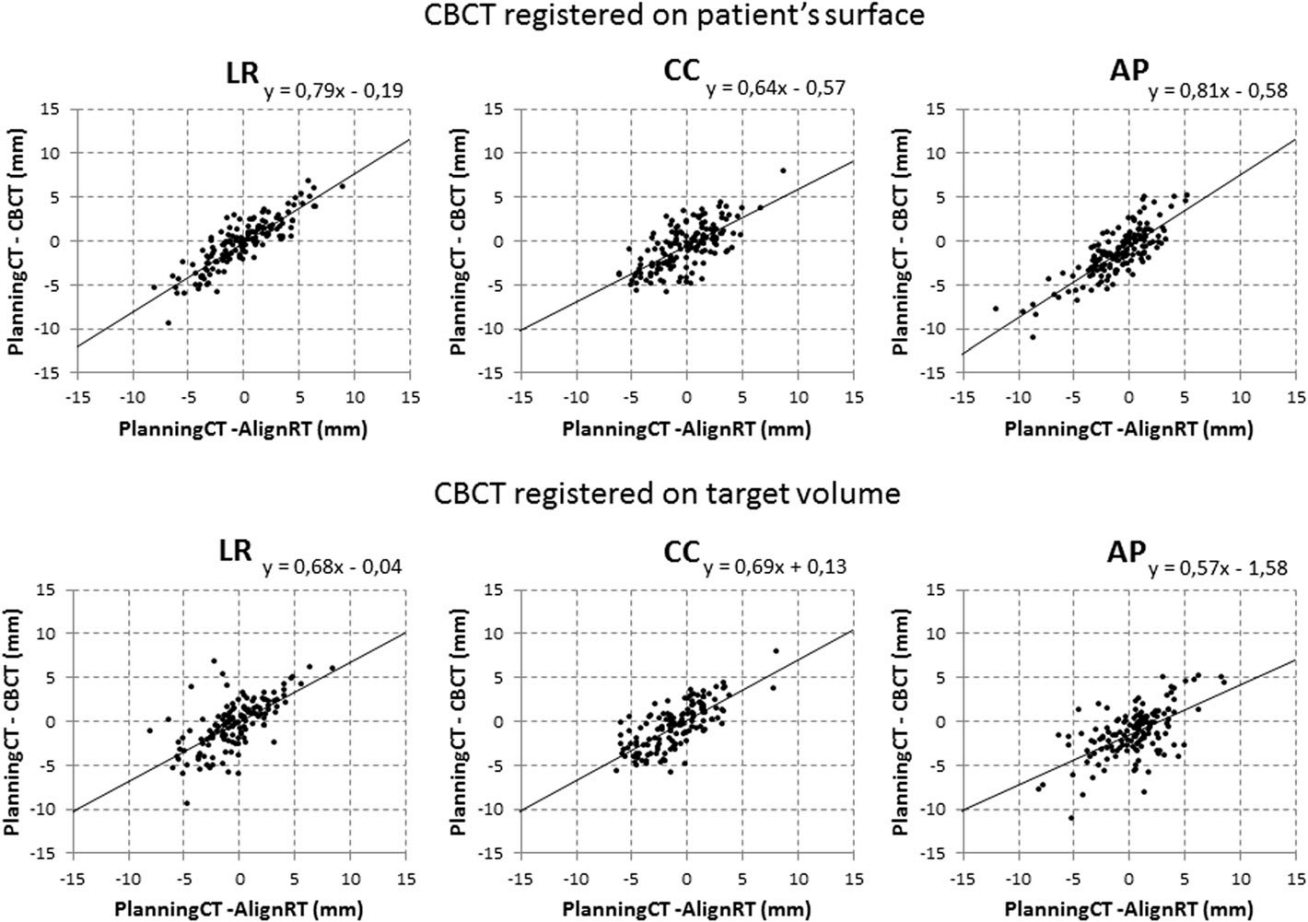
Scatterplots with regression lines of the AlignRT setup errors vs the CBCT setup errors. The corresponding correlation coefficients are given in Table 1

**Fig. 4:**
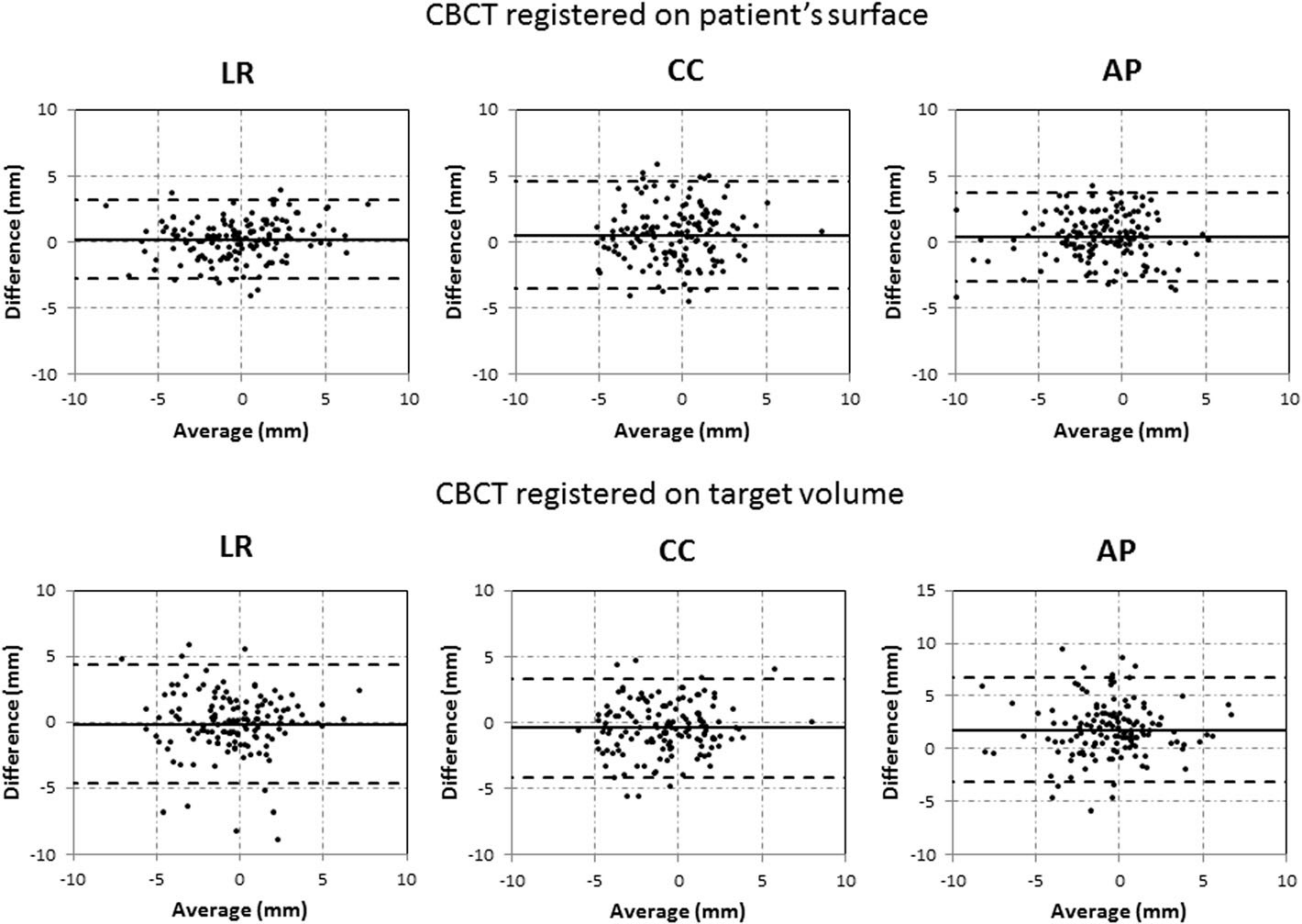
Bland-Altman plots for the setup errors of AlignRT and the CBCT registrations. The top and bottom dashed lines represent the 95% limits of agreement. The central solid line represents the mean

Table 1 lists the *M, Σ, σ, R*^*2*^ and LOA calculated for the differences in setup errors between AlignRT and CBCT target volume or surface registrations. The *Σ* and *σ* are equivalent or smaller for the CBCT surface registered results. Figure 3 shows the correlation between the AlignRT and CBCT setup errors. In Fig. 4 the Bland-Altman plots are presented for the setup errors of AlignRT and the CBCT registrations. LOA values are slightly closer when the CBCT is registered to the patient’s surface than that of the target volume, however the difference between external (surface) and internal registration methods is small (mean difference is 1.3 mm).

**Table 1:** Overview of the setup differences between AlignRT and CBCT (target volume and surface registered)

	AlignRT / CBCT target volume			AlignRT / CBCT surface		
	LR (mm)	CC (mm)	AP (mm)	LR (mm)	CC (mm)	AP (mm)
*M*	0.1	−0.5	1.7	0.1	0.6	0.4
*Σ*	1.8	1.4	1.9	1.0	1.4	1.5
*σ*	1.4	1.6	1.9	1.2	1.4	1.0
*M* - 1.96 x *SD*	−4.4	−4.3	−3.3	−2.8	−3.5	− 3.0
*M* + 1.96 x *SD*	4.5	3.2	6.6	3.1	4.7	3.8
*R* ^*2*^	0.43	0.53	0.35	0.74	0.44	0.64

*M* Group mean, *Σ* The systematic error, *σ* The random error, *M ± 1.96 x SD* The 95% limits of agreement, *R*^*2*^ The Pearson correlation coefficient, *LR* Left-right, *CC* Cranial-caudal, *AP* Anterior-posterior

For the variability and stability of the treatment BHs, a total of 1705 BHs were analyzed in 261 treatment fractions. The results for the variability and stability are shown in Table 2. The average variability was 2.4 mm and was similar in all directions. The maximum variability was 12.4 mm in the CC direction. The stability was negative in the LR direction and positive in the CC and AP direction. The absolute average stability was 1.5 mm and the maximum was 11.7 mm. Figure 2b shows sample data from one of the five excluded fractions with highly unstable BHs. During BH, the breast position increased in both the anterior and cranial directions due to a broken mouthpiece.

**Table 2:** Overview of the intra-fraction variability and stability of BHs performed with the ABC system

	LR (mm)	CC (mm)	AP (mm)	Average
Average (± 1SD)				
BH deviation with planning CT	0.2 (± 3.1)	−1.0 (± 2.6)	−1.2 (± 2.9)	−0.7 (± 2.9)
Variability	2.2 (± 1.4)	2.8 (± 1.5)	2.3 (± 1.3)	2.4 (± 1.4)
Stability	−1.0 (± 1.3)	2.1 (± 2.5)	1.5 (± 2.8)	0.9 (± 2.2)
Maximum				
Variability	7.5	12.4	6.4	
Stability	−5.8	9.6	11.7	

*BH* Breath-hold, *LR* Left-right, *CC* Cranial-caudal, *AP* Anterior-posterior

## Discussion

Multiple studies have proven the high accuracy of AlignRT corrections with respect to 2D portal imaging [19–24]. In our study 3D CBCT imaging was used to verify AlignRT corrections, indicating suitability of using SGRT for intra-fraction monitoring. Alderliesten et al. [17] also compared AlignRT to 3D CBCT imaging setup errors for DIBH radiotherapy and presented similar results. Calculated LOA were − 0.34-0.48, − 0.42-0.39, and − 0.52-0.23 cm in the left-right, cranial-caudal, and anterior-posterior directions, respectively. Their results were comparable to the LOA calculated in this study, however Alderliesten et al. used only one longitudinal camera for AlignRT monitoring.

From the LOA determined in our study, it can be concluded that 95% of the differences in set-up errors between AlignRT and the CBCT (surface registered) are within 4.7 mm. Therefore, AlignRT can replace skin markers for initial patient positioning. Other studies have already proven the benefit of AlignRT in patient positioning. Cravo Sá et al. [25] showed that positioning patients with AlignRT is more accurate than when only skin marks are used. Batin et al. [26] showed for post-mastectomy patients that positioning with AlignRT after laser alignment resulted in a significant reduction of the residual errors than when positioning with laser alignment alone.

In this study, two different CBCT registration methods were used. Although slight shading at the surface was visible on the CBCT due to the use of multiple BHs, this did not cause registration inaccuracies. This is because for the CBCT, the resulting image is an average position of the patient, and this is used for registration. The AlignRT data was also analyzed based on an average position over multiple BHs during this study, so the impact of multiple BHs is equivalent for CBCT and SGRT. At first the CBCTs were registered on the patient’s surface, as this is the region that AlignRT also uses for registration. Those results can be used to validate the registration accuracy of AlignRT. Average differences between both methods were ≤ 0.6 mm, the LOA ≤ 4.7 mm and the Σ and σ were ≤ 1.4 mm. Therefore, we concluded that AlignRT is acceptable for continuous 3D monitoring, which is not possible with CBCT. The second CBCT registration method was based on the target volume, as this is more relevant to clinical practice. The CBCTs were registered on the thoracic wall and the position errors of the clips and breast contour were maintained within 5 mm. As expected, differences from AlignRT were slightly larger than with the surface-CBCT match. However, the LOA was still within 4.5 mm for the LR and CC direction. Only for the AP direction the LOA was 6.6 mm. Therefore, we can conclude that ABC guidance can be used to maintain the target volume position of patients within 4.5 mm in the LR and CC direction in 95% of cases and within 6.6 mm in the AP direction during intra-fraction monitoring.

A higher correlation between AlignRT and the surface-CBCT data was observed for the first 3 fractions compared to the last 3 fractions (0.64 vs 0.55 respectively). This is due to the formation of lymphedema that causes changes in breast size and shape [27–29]. During the CBCT surface registration, the caudal part of the breast is mainly used for alignment in the CC direction. However, due to the ROI position, AlignRT is mainly looking at the ventral side of the breast, which can differ in shape throughout the treatment. This could explain the lower correlation between AlignRT and the CBCT surface registration in the CC direction.

The Elekta ABC was used to guide the BH. This system is spirometer-based and ensures a forced BH. The forced BH experience is uncomfortable for most patients [7, 30, 31]. The system registers the volume inhaled/exhaled by the patient with high reproducibility [32]. However, only the volume is measured, hence this does not guarantee low variability in thorax/breast positions for 100% of patients [8]. Moreover, the ABC system is not suitable for claustrophobic/anxious patients, who would benefit from an accurate, SGRT-monitored, voluntary BH method, such as AlignRT.

In this study, the intra-fraction BH position variability is on average 2.4 mm (range 2.2–2.8 mm). This result is in agreement with previous studies, where the average variability was within 0.5 mm [33], 2.2 mm [34], 1 mm [6] and 3.4 mm [35] (calculation of the variability was consistent with this study) and 4 mm (variability determined as the average standard deviation over BH positions) [36]. Other studies have also shown low variability in BH positions with the ABC system [37, 38] and other spirometer based systems [39, 40]. The stability determined in the current study is on average 0.9 mm (range − 1.0 – 2.1 mm), which is also in close agreement with Cervino et al. [41] where an average stability of 1.5 mm (range 0.1–4.2 mm) was determined with GateCT.

Although the average BH variability is small and the stability is good, outliers persist. Values up to 12.4 mm for the variability and 11.7 mm for the stability were observed in this study. This was also noticed by Moran et al. [36] who observed displacements up to 19 mm. Furthermore, errors in stability of 14.1 mm were observed for one patient caused by escape of air during the BH through a broken mouthpiece (Fig. 2b). Especially relevant for such outliers, SGRT provides additional surface displacement information that is not possible to measure with CBCT or air-volume BH systems.

The clinical target volume (CTV) to planning target volume (PTV) margin was 5 mm. In this study, the average intra-fraction deviation of the breast/thorax with respect to the planning CT in any translational direction was larger than 5 mm in 19 of the 261 treatment fractions (7.3%), which could potentially result in an under-dosage in those fractions. However, a limited effect on the total CTV dose is expected as those BH deviations were only observed in a limited number of treatment fractions per patient and a robust planning technique was used. Harron et al. [42] studied the impact on CTV coverage for a systematic 5 mm shift in all directions for breast patients. The dosimetric effect was less than 5% on the target volume receiving between the 95–107% of the prescribed dose. Moreover, Fassi et al. [35] showed only a maximum decrease of 2.1% on the CTV D_95%_ when applying rotations and translations derived from the BH variability to the original treatment plan. However, the dose gradients towards the heart and lungs remain sensitive to position variation, which can result in under-dosage to the CTV or increased dose to the heart and lungs. Planning techniques for breast radiotherapy are becoming highly modulated with increasing use of VMAT, IMRT (intensity modulated radiotherapy) or IMPT (intensity modulated proton therapy). By incorporating steeper dose gradients than ever before, low inter- and intra-fraction variability and good stability in BHs is becoming more important [43]. Robustness evaluation considering position variation as well as BH level variations is advisable, to determine the effect on the dose to the CTV.

It is expected that the clinical use of SGRT (AlignRT in this study) for DIBH treatments will result in an improved variability, stability and LOA over BH guidance based on air-volume. This is because SGRT has a similar surface accuracy to CBCT, can reliably monitor the patient surface during BH and patients must be within a certain tolerance (normally 3–5 mm), hence, large position deviations cannot occur.

## Conclusion

With SGRT, left-sided breast cancer patients can be positioned and monitored to within 5 mm with respect to reference CBCT data. The SGRT system was able to determine that a low intra-fraction variability and good stability can be achieved for most patients with the air-volume BH system. Additional patient position information is available with SGRT, that cannot be detected with CBCT or air-volume BH systems.

## Data Availability

The datasets used and/or analysed during the current study are available from the corresponding author on reasonable request.

## Abbreviations

ABC: Active breathing coordinator
AP: Anterior-posterior
BH: Breath-hold
CBCT: Cone-beam computed tomography
CC: Craniocaudal
CTV: Clinical target volume
DIBH: Deep-inspiration breath-hold
EPID: Electronic portal imaging device
FB: Free breathing
IMPT: Intensity modulated proton therapy
IMRT: Intensity modulated radiotherapy
LOA: Limits of agreement
LR: Left-right
PTV: Planning target volume
ROI: Region of interest
SD: Standard deviation
SGRT: Surface guided radiotherapy
VMAT: Volumetric modulated arc therapy
WBRT: Whole breast radiotherapy
